# In Vitro Growth of Human Keratinocytes and Oral Cancer Cells into Microtissues: An Aerosol-Based Microencapsulation Technique

**DOI:** 10.3390/bioengineering4020043

**Published:** 2017-05-14

**Authors:** Wai Yean Leong, Chin Fhong Soon, Soon Chuan Wong, Kian Sek Tee, Sok Ching Cheong, Siew Hua Gan, Mansour Youseffi

**Affiliations:** 1 Faculty of Electrical and Electronic Engineering, Universiti Tun Hussein Onn Malaysia, 86400 Parit Raja, Batu Pahat, Johor, Malaysia; joeyleongwaiyean@gmail.com (W.Y.L.); scw90@hotmail.com (S.C.W.); tee@uthm.edu.my (K.S.T.); 2 Biosensor and Bioengineering Laboratory, MiNT-SRC Research Center, Universiti Tun Hussein Onn Malaysia, 86400 Parit Raja, Batu Pahat, Johor, Malaysia; 3 Cancer Research Malaysia, 1, Jalan SS12/1A, Subang Jaya 47500, Malaysia; sokching.cheong@cancerresearch.my; 4 Human Genome Centre, School of Medical Sciences, Universiti Sains Malaysia, 16150 Kubang Kerian, Kota Bahru, Malaysia; shgan@usm.my; 5 School of Engineering, Design and Technology, Medical Engineering, University of Bradford, Bradford BD7 1DP, UK; m.youseffi@bradford.ac.uk

**Keywords:** alginate, aerosol, microencapsulation, microtissues, keratinocytes, oral squamous cell carcinoma

## Abstract

Cells encapsulation is a micro-technology widely applied in cell and tissue research, tissue transplantation, and regenerative medicine. In this paper, we proposed a growth of microtissue model for the human keratinocytes (HaCaT) cell line and an oral squamous cell carcinoma (OSCC) cell line (ORL-48) based on a simple aerosol microencapsulation technique. At an extrusion rate of 20 μL/min and air flow rate of 0.3 L/min programmed in the aerosol system, HaCaT and ORL-48 cells in alginate microcapsules were encapsulated in microcapsules with a diameter ranging from 200 to 300 μm. Both cell lines were successfully grown into microtissues in the microcapsules of alginate within 16 days of culture. The microtissues were characterized by using a live/dead cell viability assay, field emission-scanning electron microscopy (FE-SEM), fluorescence staining, and cell re-plating experiments. The microtissues of both cell types were viable after being extracted from the alginate membrane using alginate lyase. However, the microtissues of HaCaT and ORL-48 demonstrated differences in both nucleus size and morphology. The microtissues with re-associated cells in spheroids are potentially useful as a cell model for pharmacological studies.

## 1. Introduction

Monolayer cell cultures in plastic culture vessels are routinely used in biological studies. However, the use of a two-dimensional (2D) cell model has limitations in drug delivery [1,2]. In 2D culture, the proliferation, differentiation, gene and protein expression, functionality, and morphology of cells are considerably different from their physiological origin in vivo [3]. By contrast, the three-dimensional (3D) cell culture creates an artificial environment where cells are permitted to grow or interact with their surroundings. Three-dimensional cell culture was suggested to have better approximation to the tissue model for cell and tissue research as it can restores specific biochemical and morphological features similar to the corresponding tissue in vivo [4] with more native-like connections between cells [3,5].

Cutting edge biotechnology for creating living functional tissues in vitro is urgently needed for the application in cell culture and tissue engineering [6], pharmacological testing, bioengineering [7], and regenerative medicine [8,9]. Hanging drop [10] and liquid overlay [11] techniques are typically used for 3D spheroid culture. The hanging drop method is based on the sedimentation of cells by gravitational force. The volume of hanging drops is small, between 20–200 μL. For this method, it is difficult to exchange the media before mature microtissues are formed [12]. In addition, the media can evaporate easily at such a low volume [12,13]. The liquid overlay method stimulates cell-cell aggregation on a non-adherent layer on the culture plate. This method is straightforward but suffers from some shortcomings such as low reproducibility [10], production of spheroids in a wide range of sizes, and non-uniform shape of the spheroids [11,14]. 

To address issues of the previous techniques, an aerosol microencapsulation technique is proposed to produce a more uniform size of microcapsules of cells in high quantity through air flow dispersion. Microencapsulation is a research of intense interest which allows for the creation of cell and tissue models for rehabilitation of functional tissues [15] and therapeutics [16,17]. It is a technique which entraps cells within a membrane or shell with a diameter in the range of a few micrometers to several thousands of micrometers [18,19]. The semipermeable membrane of the microcapsule can facilitate the transportation of proteins, DNA, and drugs, thus allowing the diffusion of oxygen, nutrients, therapeutic products, and wastes, while blocking the entry of antibodies and immunocytes [20]. In drug delivery, microcapsules were used for loading both drugs and cells that are more cost effective in comparison to direct drug delivery [21] and, thus, overcome rejection of the implanted organ [22].

To date, various types of biopolymers including agarose, collagen, alginate, chitosan, and gelatin have been widely applied for encapsulation of cells [8,19]. Among them, alginate is the most commonly used biopolymer for encapsulation of living cells because of its many advantages [23,24]. Alginate is a naturally derived polymer, biocompatible both in vitro and in vivo, with excellent biodegradability; it provides a fast gelation process at room temperature [25]. Furthermore, alginate is recognized as a clinically ready material by the U.S. Food and Drug Administration (FDA) [20,26].

Despite its promising application, the growth of microtissue models for human skin and oral cancer cell studies is slow. Tumor spheroids provide an in vitro proliferating model for the studies of tumor growth that provides a well-defined structure of cells mimicking the microtissues of tumors [3,27]. Spheroids represent the clusters of cells usually formed by the re-association of dissociated cultured cells. The basic principle of using spheroids in 3D culture is that the cell aggregates are capable of organizing themselves into groups to form tissue-like architecture. It is now well accepted that the tumor spheroids, as a three-dimensional model, closely resembles the initial avascular stages of small solid tumors in vivo [28,29]. For normal cells, the spheroids of keratinocytes can function as a better wound healing model than the 2D wound model [30,31]. Hence, the growth of both cell types into biomimetic microtissues would be useful in tissue implant and even cancer therapeutic drugs studies. Based on an aerosol technique developed in-house, we report on the microencapsulation of the human keratinocyte (HaCaT) cell line and an oral squamous cell carcinoma (OSCC) cell line (ORL-48) in calcium alginate as a strategy to grow cell spheroids. 

## 2. Materials and Methods

### 2.1. Cell Culture and Preparation

Human keratinocyte cell line (HaCaT) was purchased from cell line services (CLS, Eppelheim, Germany) while the ORL-48 OSCC cell line was established by Cancer Research Malaysia [32,33]. HaCaT and ORL-48 cells were maintained in Dulbecco’s Modified Eagle Medium (DMEM, Gibco^®^, Life Technologies, Camarillo, CA, USA) supplemented with 10% fetal bovine serum (FBS, Invitrogen, Carlsbad, CA, USA), fungizone (2.5 mg/L, Sigma Aldrich, Dorset, UK), penicillin (100 units/mL, Sigma Aldrich, Dorset, UK), and streptomycin (100 mg/mL, Sigma Aldrich, Dorset, UK) at 37 °C in a 5% carbon dioxide (CO_2_) humidified environment. Upon reaching 80% confluency, the media was removed from the cell culture flask and the flask was washed three times with Hank’s Balanced Salt Solution (HBSS, Invitrogen, Carlsbad, CA, USA). Following removal of the HBSS solution, cells were detached from the flask with 1 mL of trypsin (0.5 mg/mL, Sigma Aldrich, Dorset, UK) and pelleted for the microencapsulation experiment.

### 2.2. Preparation of Cell-Alginate and Calcium Chloride Solutions

**Figure 1:**
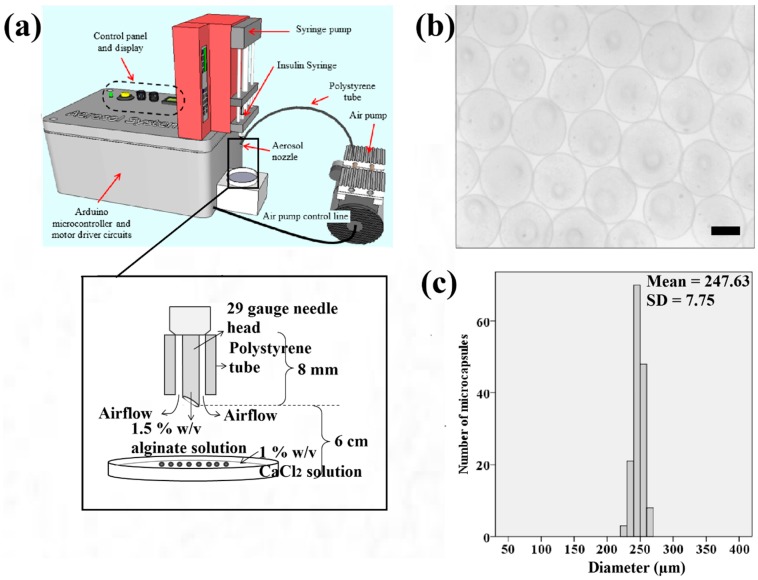
(**a**) Schematic illustration of the electronic aerosol microencapsulation system; (**b**) The photomicrographs of microcapsules (50× magnification) produced at the extrusion rate of 20 μL/min and air flow rate of 0.3 L/min (Scale bar: 200 μm); (**c**) Size distribution of the microcapsules.

Sodium alginate solution at 1.5% *wt*/*v* (Sigma Aldrich, St. Louis, MO, USA) and calcium chloride solution at 1% *wt*/*v* (Sigma Aldrich, St. Louis, Missouri, USA) were prepared by dissolving the solid crystals of sodium alginate and calcium chloride in distilled water, respectively. Both HaCaT and ORL-48 cells were added to 100 μL of alginate solution at cell densities of 3 × 10^7^ and 9 × 10^7^ cells/mL, respectively. In our experiment, ORL-48 cells did not grow further into microtissues at the lower cell density of 3 × 10^7^ cell/mL. Hence, a higher cell density of ORL-48 at 9 × 10^7^ cells/mL was applied. The cell-alginate suspension was filled into a 0.5 mL syringe (Becton, Dickinson and Company, Franklin Lakes, New Jersey, USA) with a 29-gauge insulin needle. The syringe was fitted to a syringe pump (NE-4002X, New Era, Farmingdale, NY, USA) functioned to extrude the cell-alginate suspension (Figure 1a). Subsequently, calcium chloride solution was filtered using a 0.2 μm Polytetrafluoroetylene membrane Acrodisc^®^ syringe filter (Pall^®^ Life Sciences, Port Washington, New York, USA). The needle was inserted into the air tube extending from the air pump system as shown in Figure 1a. The developed electronic aerosol system [34] (Figure 1a) was applied to extrude microdroplets of cell-alginate at an extrusion rate and air flow rate of 20 μL/min and 0.3 L/min, respectively. Filtered calcium chloride solution (4 mL) was then prepared in a sterile petri dish of 6 cm diameter for crosslinking the microdroplets of cell-alginate. The petri dish was placed 6 cm under the insulin needle as shown in Figure 1a.

### 2.3. 3D Cell Microencapsulation Using Aerosol Technique

In the aerosol microencapsulation system, HaCaT and ORL-48 cells were encapsulated in independent experiments. During the experiments, the aerosol system dispersed the microdroplets of cell-alginate from the aperture of the needle, the microdroplets dropped into the petri dish and were then polymerized in calcium alginate solution for approximately 10 min. The standard polymerization time was determined by the mean absorbance at 330 nm using Multiskan™ GO Microplate Spectrophotometer (Thermo Fisher Scientific, Waltham, MA, USA). Subsequently, the solution in the petri dish was carefully discarded, leaving only the polymerized microcapsules of HaCaT or ORL-48 cells. The microcapsules containing cells were rinsed three times with HBSS solution followed by incubation in 2 mL of DMEM at 37 °C in a 5% CO_2_ humidified incubator for 16 days. The media was replenished every two days to provide enough nutrients for the growth of the encapsulated cells. All experiments were performed in a SC2-4A1 biological safety cabinet (ESCO, Singapore). The growth of both HaCaT and ORL-48 cells encapsulated in the microcapsules were monitored every two days up to 16 days of culture. Photomicrographs of the cells were captured using an inverted phase contrast microscope (TS100, Nikon, Tokyo, Japan) coupled with a Go-5 CCD digital camera (QImaging, Surrey, UK).

### 2.4. DAPI Staining 

DAPI (4′,6-diamidino-2-phenylindole dihydrochloride) staining was performed to investigate the cells or nuclei distribution in microtissues for both HaCaT and ORL-48 following 16 days of culture. First, DAPI (0.1 μg/mL) (Sigma Aldrich, St. Louis, MO, USA) was diluted in HBSS. Both the microcapsules of HaCaT and ORL-48 were separately washed in HBSS and then incubated in DAPI solution for 20 min in the dark. Subsequently, the DAPI stain was removed and the stained microtissues were washed with HBSS solution. The stained microtissue images were viewed and captured using a BX53 fluorescence microscope (Olympus, Tokyo, Japan) mounted with a DP73 CCD camera (Olympus, Tokyo, Japan).

### 2.5. Live and Dead Cell Staining

The live/dead^®^ viability kit for mamalian cells (Invitrogen, Paisley, UK) was used to stain the live and dead cells of the microtissues formed in the calcium alginate microcapsules. The live/dead^®^ cell viability kit can differentiate live cells from the dead cells by double staining of both the HaCaT and ORL-48 cells in the microtissues with green-fluorescent Calcein-Acetoxymethyl (AM, Invitrogen, Paisley, UK), which indicates intracellular esterase activity, and red-fluorescent Ethidium homodimer-1 (EthD-1) (Invitrogen, Paisley, UK), which indicates the loss of plasma membrane integrity. After 16 days of culture, the HaCaT and ORL-48 microtissues formed in the calcium alginate microcapsules were incubated in 2 μM of Calcein-AM and 4 μM of EthD-1 stain solutions for 20 min in the dark. Subsequently, the stain solutions were removed and the microtissues were washed three times in HBSS solution. The stained microtissues were captured using a BX53 fluorescence microscope (Olympus, Tokyo, Japan) mounted with a DP73 digital camera. 

### 2.6. Alginate Lyase Activity

The alginate shell of the microcapsules of HaCaT and ORL-48 were removed by using alginate lyase (Sigma Aldrich, St. Louis, MO, USA) at 0.2 mg/mL prepared in a media after 16 days of culture. Alginate lyase catalyzes the biodegradation of complexly structured alginate by cleaving the glycosidic bond via a β-elimination reaction [35]. Within 1 to 2 min of immersion in the alginate lyase media, the 3D microtissues released from the alginate shell were collected and washed three times in HBSS. The purified microtissues were ready for physical examination using a field emission-scanning electron microscopy (FE-SEM).

### 2.7. Field Emission-Scanning Electron Microscopy

The shapes, external morphology, and surface structure of both HaCaT and ORL-48 microtissues were examined by using a JSM-7600F field emission-scanning electron microscope (FE-SEM, JOEL, Tokyo, Japan) with an upper secondary electron imaging (SEI) detector. Before imaging, the microtissues of HaCaT and ORL-48 were fixed in 4% formaldehyde (Sigma Aldrich, St. Louis, MO, USA) for 24 h at 5 °C. The fixed microtissues were then transferred and placed on 2 × 2 cm microscope glass slides before being left to air dry at room temperature. Subsequently, the glass slides containing the 3D microtissues were coated with a conductive gold coating in a JFC-1600 Auto Fine Coater (JOEL, Tokyo, Japan) powered at 20 mA for 30 s. Then, the gold coated glass slide containing the microtissues was mounted to a mounting stub using double-sided carbon tapes before loading into the FE-SEM for imaging. During the FE-SEM scanning, both the microtissues of HaCaT and ORL-48 were exposed to an accelerated voltage beam at 5 kV. The reflected beam from the samples was detected using an upper secondary electron imaging (SEI) detector.

### 2.8. Re-Plating Microtissues

Re-plating of microtissues is our newly proposed method to investigate the physical changes in the microtissues on a culture dish and examine the viability of the microtissues. The microtissues of HaCaT and ORL-48 were extracted from the calcium alginate microcapsules and re-plated in a petri dish. The released 3D microtissues from the calcium alginate microcapsules were washed three times in HBSS and then transferred to two independent petri dishes with a diameter of 35 mm. Two mL of DMEM was then added into the petri dish. The physical changes in the microtissues following the removal of the alginate shell were monitored every 24 h up to 72 h. The effects of re-plating 3D microtissues were observed and captured using a TS100 inverted phase contrast microscope (Nikon, Tokyo, Japan) that was linked to a Go-5 CCD digital camera (QImaging, Surrey, UK).

## 3. Results

Figure 1b shows that the samples of microcapsules produced in spherical shape using the aerosol technique. The microcapsules of calcium alginate have a narrow size distribution ranging from 220 to 270 μm as indicated (Figure 1c). This range falls within the recommended ideal size of microcapsules for epithelial cells to grow to the approximate thickness of the epidermis which is between 200 to 300 μm.

**Figure 2:**
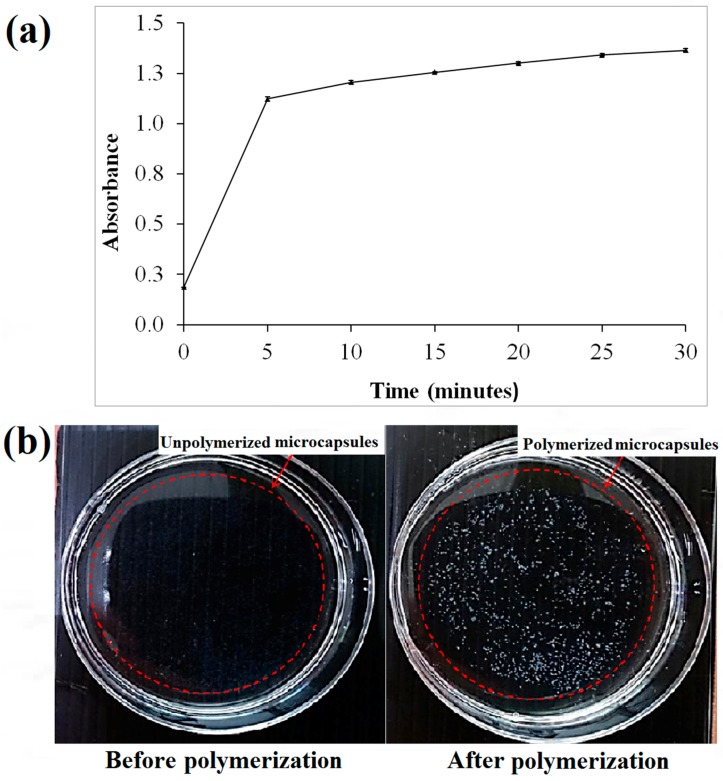
(**a**) The polymerization absorbance of the microcapsules upon irradiation at 330 nm; (**b**) Images of calcium alginate microcapsules before and after polymerization.

Based on the spectrophotometry of the microcapsules (Figure 2a), the threshold time for the polymerization of alginate was approximately 5 min following the immersion of the sodium alginate in the calcium chloride solution. This was based on the drastic change of absorbance at approximately 5 min after polymerization (Figure 2a). Hence, it was suggested to polymerize the microcapsules of alginate with at least 5 min of immersion in the calcium chloride solution. The minimum period for polymerization was studied to ensure the microcapsules produced have stiffness in the range of approximately 136 kPa making them suitable for cell culture [36]. The un-polymerized alginate microcapsules are transparent in comparison with cross-linked alginate microcapsules that appeared white (Figure 2b).

**Figure 3:**
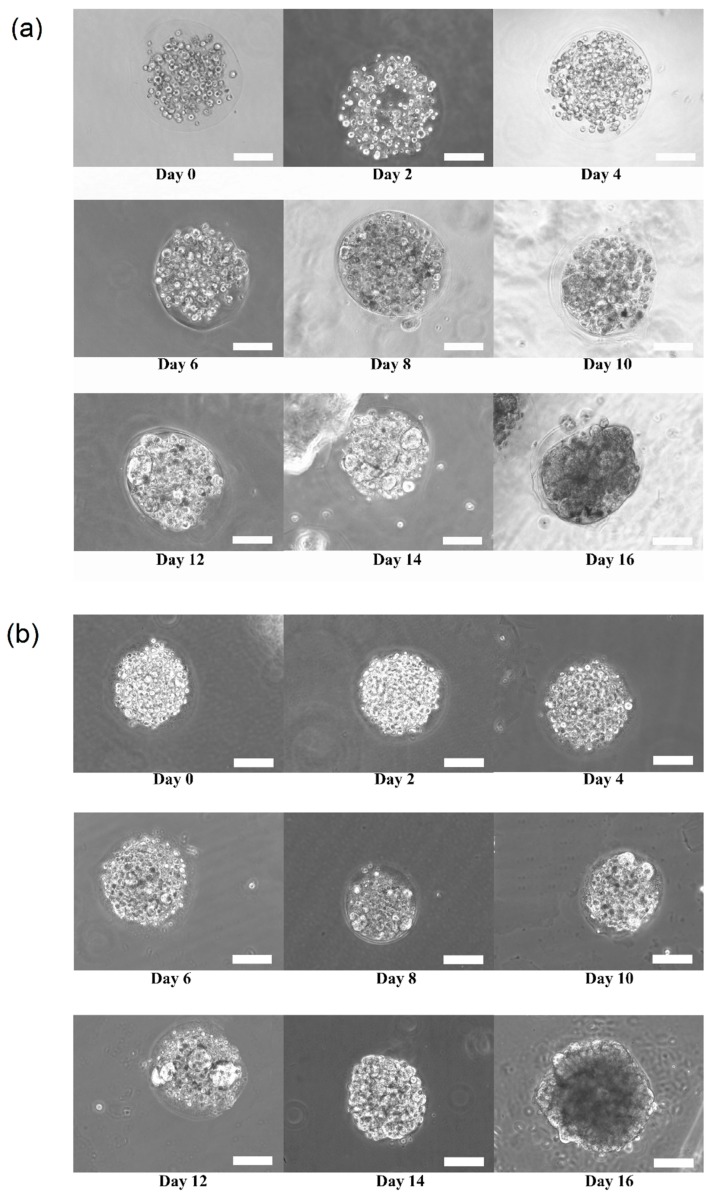
Phase contrast microscopic image of calcium alginate encapsulated (**a**) Three-dimensional (3D) human keratinocytes (HaCaT) and (**b**) oral squamous cell carcinoma (OSCC) cell line (ORL-48) growth for 16 days of culture at 100× magnification (Scale bar: 100 μm).

Figure 3a,b shows the growth and transition of morphological changes of 3D HaCaT and ORL-48 cells in the microencapsulation of calcium alginate over a period of 16 days, respectively. The 3D cells of HaCaT and ORL-48 cells were observed to have grown gradually into the microtissues after approximately 16 days of culture. On the first day of culture (Day 0), the HaCaT and ORL-48 cells (Figure 3) were scattered in the calcium alginate microcapsules. From Day 2 onwards, the quantity of cells encapsulated in the calcium alginate microcapsules increased and continued to form aggregates in the encapsulations (Figure 3). After approximately 14 days of culture, the clusters of cells in the microcapsules grew into microtissues and the masses of cells completely filled the microcapsules. Towards day 16 of culture, the grown microtissues of HaCaT and ORL-48 can be clearly seen with dark appearance representing a high density of microtissues.

**Figure 4:**
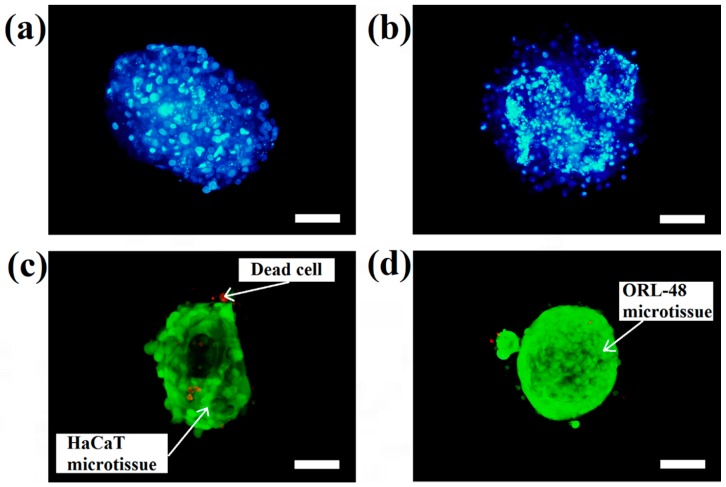
DAPI (4′, 6-diamidino-2-phenylindole dihydrochloride) staining of cells in the microtissues of (**a**) HaCaT and (**b**) ORL-48 after 16 days of culture at 100× magnification (Scale bar: 100 μm). Live and dead staining fluorescence microscopic images of (**c**) HaCaT and (**d**) ORL-48 microtissues after 16 days of culture at 100× magnification (Scale bar: 100 μm).

The micrographs of DAPI staining indicate that the distributions of nuclei for both cell types are highly concentrated in the microcapsules following 16 days of culture. These results indicated that HaCaT (Figure 4a) and ORL-48 (Figure 4b) were tightly organized in the microcapsules. However, ORL-48 cells had smaller nuclei as compared to that of HaCaT from a similar size of microcapsules following 16 days of culture. The HaCaT and ORL-48 cells in the microtissues were distinguishable in terms of size and shape, which may be caused by the often highly variable size of cancer [36]. In addition, the nucleus of cancer cells in the tissue is usually an abnormal shape due to damaged or altered genes [37]. The ORL-48 cells had a stronger stain of DAPI when compared with that of HaCaT cells (Figure 4a,b), although similar camera sensitivity was used. A previous report [38] revealed that the stronger DAPI stain observed in cancer cells may be due to their excess DNA contents. Figure 4c,d show the results of live and dead cell stainings for both the HaCaT and ORL-48 microtissues following 16 days of culture, respectively. The calcium alginate microcapsule membrane is semipermeable for the live/dead stain to allow permeation into the core of the encapsulated microtissues. The green (Calcein-AM) and red (Ethidium homodimer-1) fluorescence staining indicated live and dead cells within the microtissues, respectively. Both microtissues revealed a high viability of cells.

**Figure 5:**
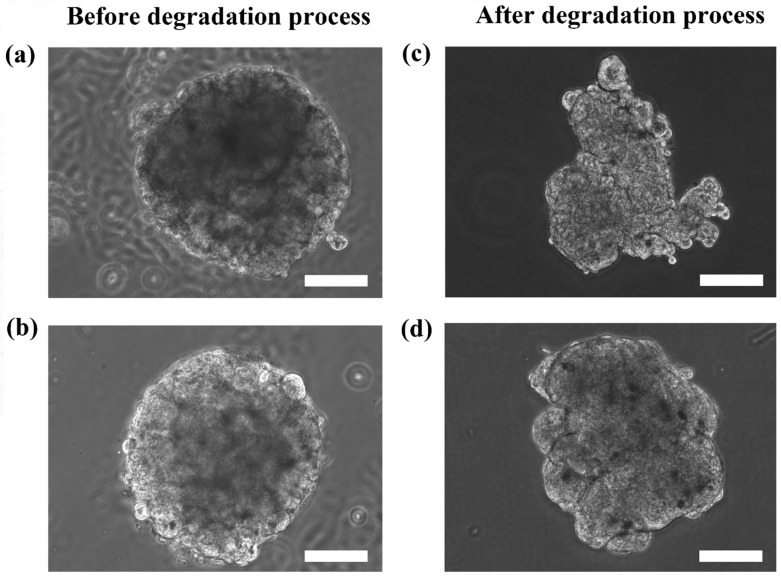
Phase contrast microscopic images of the calcium alginate encapsulated with (**a**) HaCaT and (**b**) ORL-48 microtissues before alginate degradation, and the extracted (**c**) HaCaT and (**d**) ORL-48 microtissue after alginate degradation at 100× magnification (Scale bar: 100 μm).

Although the microtissues were confined in the spherical structure of the microcapsules, it is interesting to determine if the microtissues that were formed were also indeed spherical. Figure 5 shows the HaCaT and ORL-48 microtissues before (Figure 5a,b) and after (Figure 5c,d) removal from the alginate capsules, respectively. The calcium alginate microcapsule membrane disappeared completely within 1 to 2 min after immersion in the media containing alginate lyase at 0.2 mg/mL. Both the extracted microtissues of HaCaT (Figure 5c) and ORL-48 (Figure 5d) had good cell-cell integrity and cell adhesions. No loose pieces of microtissues were observed even after re-immersion in baths of different media. However, the microtissues of HaCaTs were not able to maintain a spherical shape after a few rinsings with HBSS solution (Figure 5c). 

**Figure 6:**
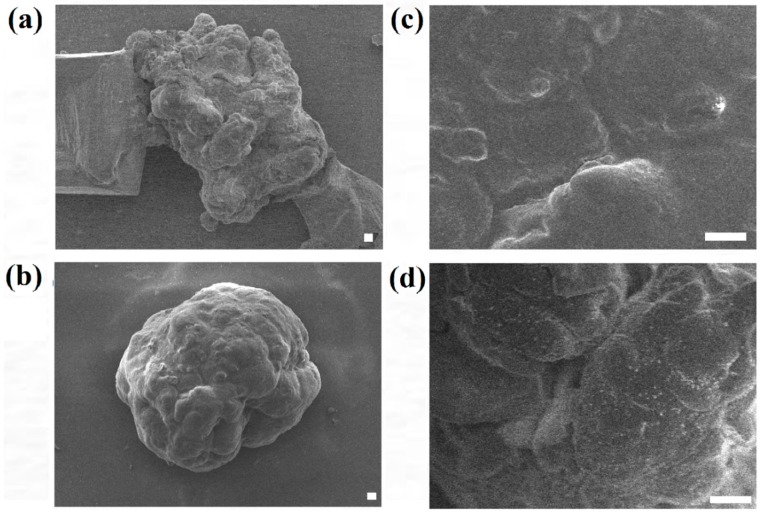
Field emission-scanning electron micrographs of (**a**,**c**) HaCaT and (**b**,**d**) ORL-48 microtissue after 15 days of culture at (**a**,**b**) 300× and (**c**,**d**) 1500× magnifications, respectively (Scale bar: 10 μm).

In order to further analyze the surface morphology of the HaCaT and ORL-48 microtissues collected, FE-SEM was applied to investigate the overall and surface structure of the microtissues. Figure 6 shows the FE-SEM micrographs of HaCaT and ORL-48 microtissues after 15 days of culture at 300× and 1500× magnifications, respectively. HaCaTs and ORL-48 (Figure 6a,b) both had good integrity of cells. However, ORL-48 microtissues appeared to be spherical in shape with a homogeneous surface (Figure 6b,d), which was probably due to the binding by self-induced extracellular matrix (ECM) proteins. A previous work [39] revealed that the surface of the human liver cancer (HepG2) microtissue is similarly homogeneous with tight cell-cell adhesions. 

**Figure 7:**
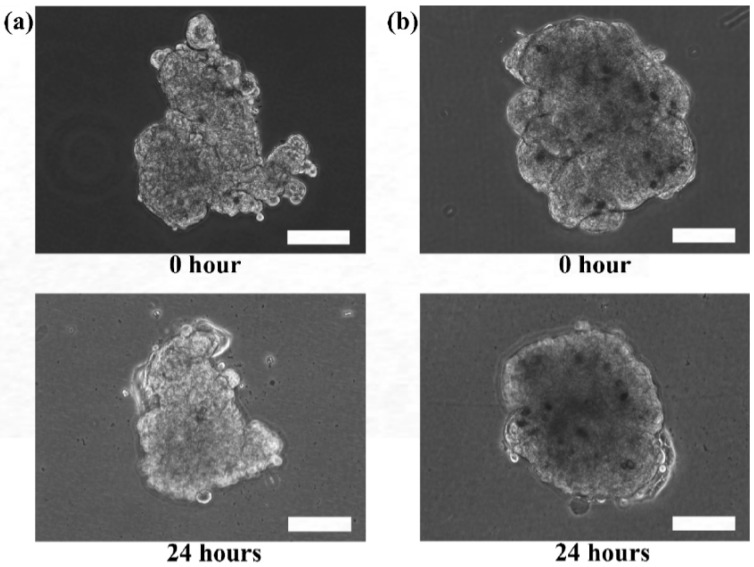

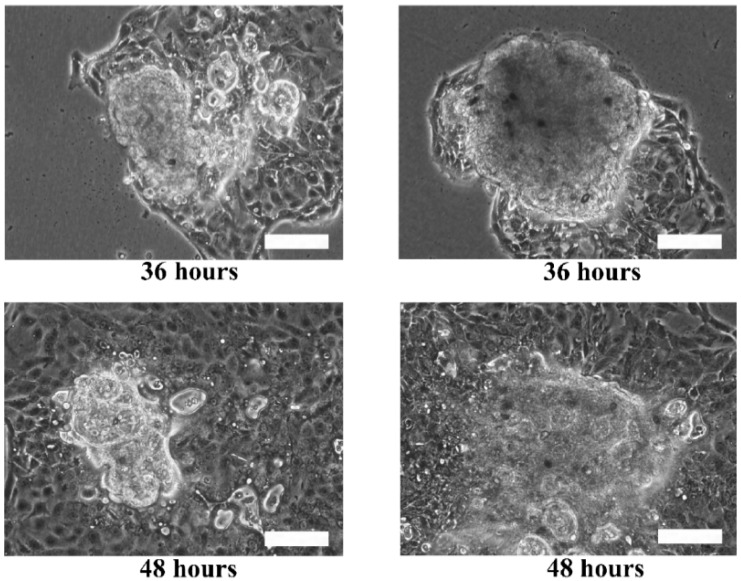
Phase contrast microscopic images of re-plating the (**a**) HaCaT and (**b**) ORL-48 microtissues at 100× magnification (Scale bar: 100 μm).

Although the live and dead cell staining suggested that the cells in the microtissues were viable, the technique could not reveal the basic functionalities of the cells, such as motility. The re-plating experiment was performed to verify the functionality of the cells during migration and proliferation in order to ensure that the cells retain their functions when they are still viable. Hence, the extracted HaCaT and ORL-48 microtissues were then re-seeded in a petri dish to investigate their physical changes (Figure 7). Several hours after transferring the microtissues of HaCaT (Figure 7a) and ORL-48 (Figure 7b) to a tissue culture-treated dish, the adhesion of the microtissues was examined by a mild perturbation of media by gently shaking the media. The microtissues were found to be well adhered to the surface of the petri dish. After 24 h of re-plating, the individual cells were found to be migrating out of the microtissues (Figure 7a,b). As more and more cells migrated out of the microtissues after 48 h of culture, this induced a monolayer of cells to form around the microtissues. The 2D monolayer of cells continued to proliferate with a larger covered area in the petri dish while the 3D microtissues gradually disintegrated after 72 h of culture (Figure 7a,b). This indicates that the cells preferably attached to a tissue culture treated dish. 

## 4. Discussion

In microencapsulation of cells, the size of the microtissues produced using the aerosoling method is in the range of 220–270 um and these microspheroids were cultured in a large volume of culture media. The aerosol microencapsulation technique produces microtissues that are easy to handle and allows for the exchange of media every day while the cell aggregates are growing. In comparison to hanging drop and overlay techniques [10], the aerosole method produces a high number of microspheroids with controlled size and shape [34]. Microcapsules within a few hundreds of diameter could reduce the mass transfer resistance by providing greater surface area for the diffusion of oxygen and nutrients to the cells located at the core of the microcapsules [40]. The cells encapsulated in the hydrogel-like capsule received sufficient nutrients and gases which allow them to stay viable after 16 days of culture. A recent work [11] based on the hanging drop method showed that 7-day old spheroids with a size of approximately 500 μm were observed with central necrosis. This report indicates that in culture, large microtissues that contain a small volume of media droplets are liable to high metabolism and nutrient deprivation of cells in the center of spheroid [11,41]. 

The confinement of cells in microcapsules ensures close proximity of 3D cells with reduced cell-cell distance in an enclosed environment, which is an important factor to stimulate the growth of cells into microtissues [42]. However, the proliferation of cells seemed to be limited by the volume of alginate microcapsules, and they remained quiescent without enlargement of size as observed after formation of microtissues. This is similar to the culturing of monolayer of cells to full confluency, in which the proliferation is limited by the size of the culture flask. In the culture media, the color change of phenol red (from red to yellow) in the presence of the microcapsules of cells indicated an exchange of catabolites and gases from the cells leading to a decrease of pH in the media. Additionally, other factors, including the soluble factors in the media and the type of biopolymer used, may also affect the growth of microtissues. However, the cell density is also a crucial factor for different cell types to grow into microtissues. In this work, ORL-48 cells at a lower density of 3 × 10^7^ cells/mL were unable to grow into microtissues but were characterized with a few aggregations of cells in the microcapsules. This could be due to the variation of cell growth rates for different cell types [43]. Hence, the growth of cells in micro-encapsulations is influenced by the size of microcapsules, cell density, cell-cell interactions within the confinement, and rigidity of the growth environment. 

Figure 4c,d indicate that both cell types in the microtissues were viable after 16 days of culture since only a few HaCaT cells were stained in red (Figure 4c). This result indicated that the cells encapsulated in the hydrogel-like microcapsules received sufficient nutrients and gases which allow them to stay alive even after long period of culture. Nevertheless, the alginate lyase showed no cytotoxicity to the microtissues.

The results showed that the microtissues may appear as spheroids due to the shape of the microcapsules. To reveal the actual appearance of the microtissues without disrupting the structure of the microtissues, alginate degradation was performed within a short period of time (1–2 min) to reduce the viscosity of the alginate capsule. The microtissues after alginate degradation can be in spheroids or non-spheroids depending on the self-organization ability of cells to form microtissues as shown in Figure 5.

Between the microtissues of the two cell types, microtissues of ORL-48 retained their spheroidal shape suggesting that cancer cells possess strong organization ability and adhesion properties. The difference in the 3D structure restorations of HaCaT and ORL-48 suggested that cancer cells might produce adequate extracellular matrix proteins which support good cell-cell adhesion, as observed in FE-SEM microscopy. 

Growing cell spheroids based on the microencapsulation technique has the advantages of spheroid size control and re-association of cells based on self-generated extracellular matrix proteins. HaCaTs and ORL-48 have the ability to self-assemble into microtissues if they are provided with a suitable and biocompatible micro-environment such as alginate based microcapsules. The multicellular structure serves as a protective layer for diffusion of molecules from the outer surface to the center core of microtissues. In this work, we did not observe uncontrollable reproduction of cancer cells as reported in the literature [44], but both cells formed microtissues well within 16 days of culture. The morphology of cells, cell-cell adhesion, cell-matrix interactions, and volumetric growth of microtissues are distinctly different from 2D culture. Thus, making the microtissue a translational model for studying the efficacy of drugs is fundamental to inform in vivo animal studies. For implementation in tissue transplantation, the microcapsular membrane is a layer which protects the microtissues from the immune system of the recipients [45]. However, the membrane can be easily removed if it is not needed, as demonstrated in this work.

The re-plating experiments verified that the microtissues were composed of viable living cells that could adapt and migrate in the culture dish. Furthermore, the surface of tissue culture treated dishes is usually modified to be more hydrophilic by generating negatively charged hydroxyl groups on the surface. The increase of negative charges on the surface of the culture dish would change the surface properties of the dish [46]. This could attract cells to migrate from the microtissues to the surface with higher affinity to the cells. 

## 5. Conclusions

The microencapsulation of HaCaT and ORL-48 cells leading to the growth of microtissues based on the aerosol technique was successfully demonstrated in this paper. The polymerization time for the alginate requires a minimum of 5 min following the immersion of sodium alginate in calcium chloride solutions. The feasibility of cell-enclosing microcapsules as a scaffold for microtissues growth within 16 days of culture was also revealed. The encapsulated cells had the ability to proliferate, self-assemble, and grow into microtissues in the microcapsules of calcium alginate. Larger size of cell nuclei was observed in HaCaT microtissues as compared to that in the ORL-48 microtissues, while ORL-48 cells exhibited much more variability in cell size. The surface structure of the ORL-48 microtissues was expressed with lower surface roughness when compared with that of HaCaT microtissues. Degradation of calcium alginate membrane using alginate lyase provided for in vitro 3D microtissues model. The microtissues re-plating experiment is a suitable test to examine the basic cell functionality of the microtissues.
